# A multi-data fusion deep learning model for prognostic prediction in upper tract urothelial carcinoma

**DOI:** 10.3389/fonc.2025.1644250

**Published:** 2025-08-06

**Authors:** Hongdi Sun, Siping Chen, Yongxing Bao, Fengyan You, Honghui Zhu, Xin Yao, Lianguo Chen, Jiangwei Miao, Fanggui Shao, Xiaomin Gao, Binwei Lin

**Affiliations:** ^1^ Department of Hematology, The Third Clinical Institute Affiliated to Wenzhou Medical University (Wenzhou People’s Hospital), Wenzhou, Zhejiang, China; ^2^ Department of Urology, Rui’an People’s Hospital, The Third Affiliated Hospital of the Wenzhou Medical University, Wenzhou, Zhejiang, China; ^3^ Department of Urology, The First Affiliated Hospital of Wenzhou Medical University, Wenzhou, Zhejiang, China; ^4^ Department of Pharmacy, The First Affiliated Hospital of Wenzhou Medical University, Wenzhou, Zhejiang, China; ^5^ Key Laboratory of Diagnosis and Treatment of Severe Hepato-Pancreatic Diseases of Zhejiang Province, The First Affiliated Hospital of Wenzhou Medical University, Wenzhou, Zhejiang, China; ^6^ Department of Laboratory Medicine, The First Affiliated Hospital of Wenzhou Medical University, Wenzhou, Zhejiang, China; ^7^ Department of Clinical Laboratory, Key Laboratory of Clinical Laboratory Diagnosis and Translational Research of Zhejiang Province, The First Affiliated Hospital of Wenzhou Medical University, Wenzhou, Zhejiang, China

**Keywords:** upper tract urothelial carcinoma, deep learning, prognostic indicators, CT image, multi-phase contrast-enhanced CT, clinical data, artificial intelligence, radiomics

## Abstract

**Background:**

Upper tract urothelial carcinoma (UTUC) is a rare but highly invasive urinary malignancy with a high postoperative recurrence rate.

**Methods:**

We retrospectively collected data from 133 UTUC patients who underwent radical nephroureterectomy between 2005 and 2017. Patients were divided into a training set (n=103) and a testing set (n=30). A multi-modal deep learning model named Multi-modal Image-Clinical Combination Classifier (MICC) was developed by integrating multi-phase contrast-enhanced CT imaging and clinical data. The model’s prognostic performance was compared with two unimodal models—ImageNet (CT-based) and ClinicalNet (clinical data-based)—and traditional clinical parameters including pathological T stage. Feature importance was evaluated using SHapley Additive exPlanations (SHAP).

**Results:**

The MICC model achieved superior prognostic accuracy with AUCs of 0.918 and 0.895 in the training and testing sets, respectively, outperforming unimodal models. Classification metrics were robust, with accuracy of 0.854, sensitivity of 0.889, specificity of 0.836, negative predictive value (NPV) of 0.933, and positive predictive value (PPV) of 0.744. Precision-recall analysis confirmed strong identification of high-risk patients despite dataset imbalance. SHAP analysis highlighted that CT imaging features contributed most significantly to the model’s predictions.

**Conclusion:**

Integrating multi-phase CT imaging with clinical data, the MICC model provides accurate prognostic prediction for UTUC patients. This approach has potential to assist clinicians in personalized risk stratification and treatment planning, ultimately improving patient outcomes.

## 1 Introduction

Upper tract urothelial carcinoma (UTUC) is a rare malignancy, accounting for approximately 5% to 10% of all urothelial carcinomas (1, 2). Prognosis in UTUC is closely linked to tumor grade and stage, with higher-grade tumors associated with significantly reduced 5-year survival rates (3, 4). Accurate identification of high-risk tumors is essential for formulating effective long-term treatment strategies (5). Therefore, the development of precise prognostic models is critical to support urologists in risk stratification and clinical decision-making.

Previous prognostic models have largely overlooked the prognostic value of CT imaging. Contrast-enhanced CT, the primary imaging modality for UTUC diagnosis, offers superior visualization of tumor characteristics, including size, shape, enhancement patterns, and local invasion, as well as involvement of adjacent structures such as renal parenchyma, vessels, and lymph nodes (6, 7). However, the diagnosis of tumor by contrast-enhanced CT mainly relies on the experience of the radiologist. Therefore, there is a possibility of missed diagnosis, especially for early-stage or small tumors.

Deep learning (DL) has shown substantial promise in prognostic modeling across various malignancies, including colorectal (8, 9), lung (10), and liver cancers (11). However, its application in predicting outcomes for UTUC remains largely unexplored. A key advantage of DL lies in its ability to autonomously extract complex and clinically relevant features from high-dimensional, heterogeneous data with minimal human intervention (12, 13). This makes DL particularly well-suited for the analysis of CT imaging, which is inherently noisy and variable. Therefore, DL presents an opportunity for more accurate and innovative risk stratification for patients with UTUT (14).

Our study introduces several key technical innovations that distinguish it from existing UTUC research. Unlike traditional radiomics approaches that rely on manual ROI delineation and hand-crafted features (15), our RGBA fusion method enables end-to-end automatic feature learning directly from multi-phase CT images. This approach overcomes the limitations of single-phase deep learning methods (16) by preserving complete temporal dynamics across all contrast phases. Furthermore, our Multi-modal Image-Clinical Combination Classifier (MICC) advances beyond conventional statistical methods (nomograms, Cox regression) (17, 18) by leveraging deep neural networks to capture complex, non-linear interactions between imaging and clinical features without requiring explicit feature selection or dimensionality reduction.

In this study, we propose a novel multi-modal DL model, termed the Multi-modal Image-Clinical Combination Classifier (MICC), which integrates multi-phase contrast-enhanced CT imaging with clinical variables to predict postoperative prognosis in UTUC patients. This approach has the potential to improve predictive accuracy and quality of information available for individualized clinical decision-making.

## 2 Materials and methods

### 2.1 Patients

This study was reviewed and approved by the Ethics Committee on Clinical Research of The First Affiliated Hospital of Wenzhou Medical University (Approval No. KY2023-R165; Approval Date: August 14, 2023). Given the retrospective design, the requirement for informed consent was waived.

**Figure 1:**
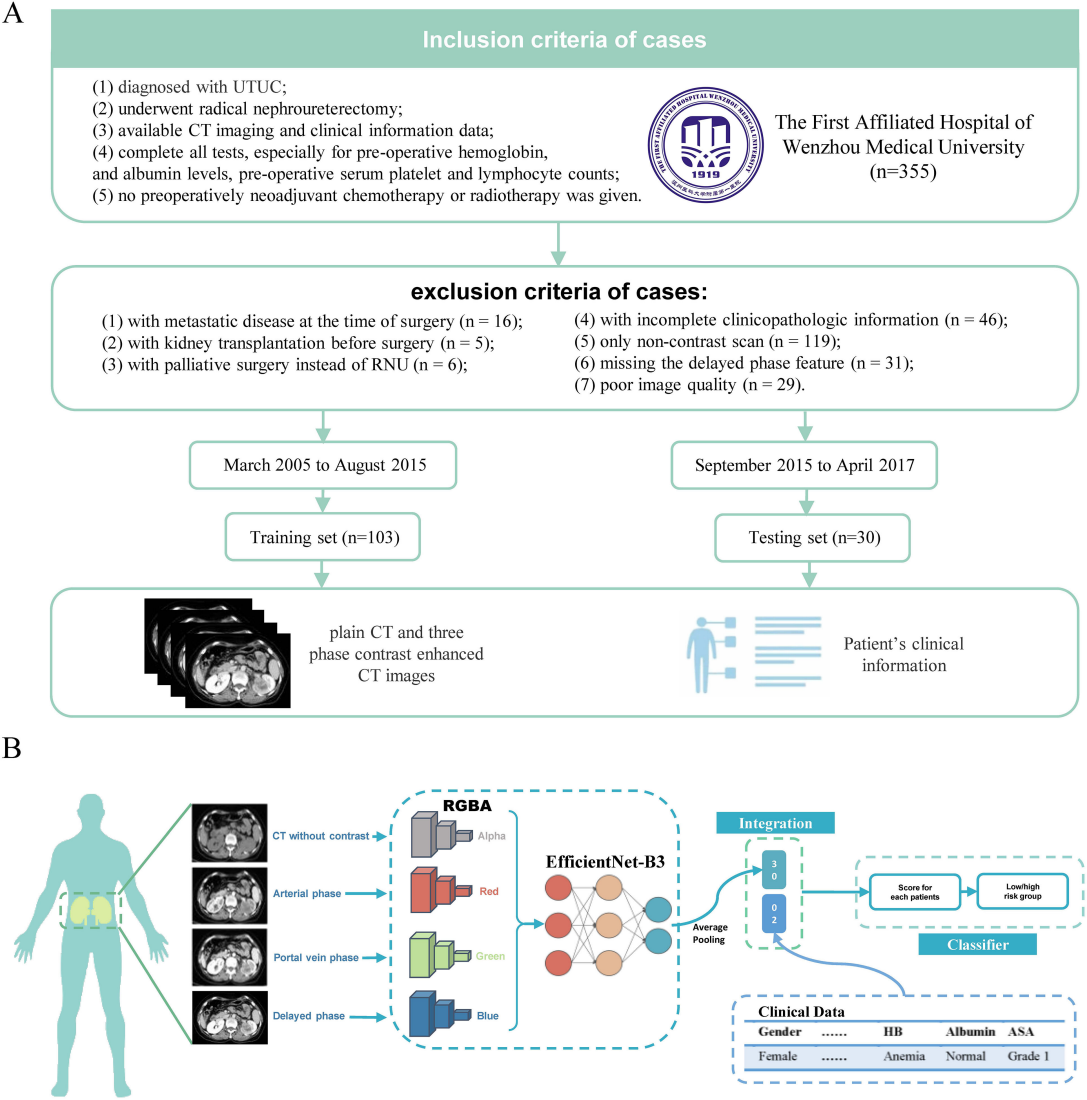
processing of the MICC model and patient inclusion/exclusion criteria. **(A)** The MICC model processes CT images stored as DICOM files, reducing noise and resizing images. It transforms the four phases of CT (arterial phase, portal vein phase, delayed phase, and plain scan) into RGBA (Red, Green, Blue and Alpha) arrays, which are then consolidated into color images. These RGBA images are input into a model based on EfficientNet-B3, and are subjected to average pooling, integrated with clinical data. A cross-entropy loss function is used to train the model, generate scores for each patient, and categorize patients into high and low-risk groups based on the optimal cutoff value from the ROC analysis. **(B)** Out of 355 patients from the First Affiliated Hospital of Wenzhou Medical University, 133 were enrolled in this study and divided into the Training set (n=103) and Testing set (n=30). Both CT images and clinical information were included in this study.

We conducted a retrospective analysis of medical records from patients who underwent contrast-enhanced CT for UTUC at our institution between March 2015 and April 2017. Inclusion and exclusion criteria for the 133 eligible patients are detailed in **Figure 1A**.

Clinical variables collected for each patient included: gender (female *vs*. male), age (≥65 *vs*.<65 years), body mass index (BMI ≥25 *vs*.<25 kg/m²), American Society of Anesthesiologists Physical Status Classification (ASA ≥3 *vs*.<3), presence of hydronephrosis (yes *vs*. no), surgical approach (open *vs*. laparoscopic), hemoglobin (Hb) levels, albumin levels, tumor size (>3 cm *vs*. ≤3 cm), tumor location (renal pelvis *vs*. ureter *vs*. both), tumor multiplicity (multiple *vs*. unifocal), lymphovascular invasion (LVI: yes *vs*. no), tumor stage (pT1–pT2 *vs*. pT3–pT4), and nodal stage (N0 *vs*. N1).

Postoperative follow-up included cystoscopy, computed tomography (CT) imaging, and both urine and blood tests. Patients were followed every three months during the first year and annually thereafter. Outcomes were defined as follows: overall survival (OS) was the interval from surgery to death from any cause; cancer-specific survival (CSS) was the interval from surgery to death specifically due to UTUC; and progression-free survival (PFS) was defined as the time from surgery to either tumor recurrence or death.

### 2.2 Study design and data partitioning

To ensure methodological rigor and prevent data contamination, a strict data separation protocol was implemented. The 133 patients were randomly assigned to a Training set (n = 103) and an independent Testing set (n = 30) using stratified sampling to preserve the distribution of outcome classes across both sets—an essential consideration given the class imbalance inherent in survival data.

Within the Training set, five-fold cross-validation was employed for model development and hyperparameter tuning. In each fold, 80% of the data were used for model training and 20% for validation. This cross-validation strategy enabled parameter optimization while mitigating the risk of overfitting. All feature extraction, data normalization, and model architecture design were conducted exclusively within the Training set, with no access to the Testing set at any stage of model development.

Three models were developed: the Multi-modal Image-Clinical Combination Classifier (MICC), ImageNet, and ClinicalNet. All models were fully trained and finalized using only the Training set and the cross-validation procedure. Model architectures, hyperparameters, and configurations were determined prior to Testing set evaluation, ensuring complete isolation of the test data.

The Testing set was reserved strictly for final model evaluation and was accessed only once, after all model parameters were fixed. This rigorous separation eliminated the possibility of data leakage and enabled an unbiased evaluation of each model’s generalizability to previously unseen cases. Notably, the MICC model’s superior performance on the Testing set was identified during this single final evaluation, rather than through iterative testing or model selection based on Testing set outcomes.

To ensure fair and direct comparisons, all three models—MICC, ImageNet, and ClinicalNet—were trained using the same data partitioning protocol, including the identical Training set (n = 103) for cross-validation and the same independent Testing set (n = 30) for final performance assessment. This consistent methodology allowed for valid comparisons across models utilizing different input modalities.

### 2.3 Preprocessing of CT images

The preprocessing workflow for the MICC model is illustrated in **Figure 1B**. All CT images, including contrast-enhanced and non-enhanced scans, were stored in DICOM (Digital Imaging and Communications in Medicine) format. To minimize background noise from adjacent organs, the window width and level were standardized at 300 and 40, respectively. From each 3D CT scan, only the 2D axial slice showing the largest tumor cross-section along the z-axis was selected for model development.

All selected images were resized using OpenCV to conform to the input dimensions required by the model. Intensity normalization was performed by computing the mean and standard deviation for each image channel. Each phase of grayscale CT imaging was then transformed into a four-channel RGBA image using NumPy: the plain, arterial, portal venous, and delayed phases were assigned to the alpha, red, green, and blue channels, respectively (19). The resulting multi-phase images were fused into a single RGBA composite image for subsequent feature extraction and modeling.

### 2.4 Feature extraction and fusion via RGBA color transformation

**Figure 2:**
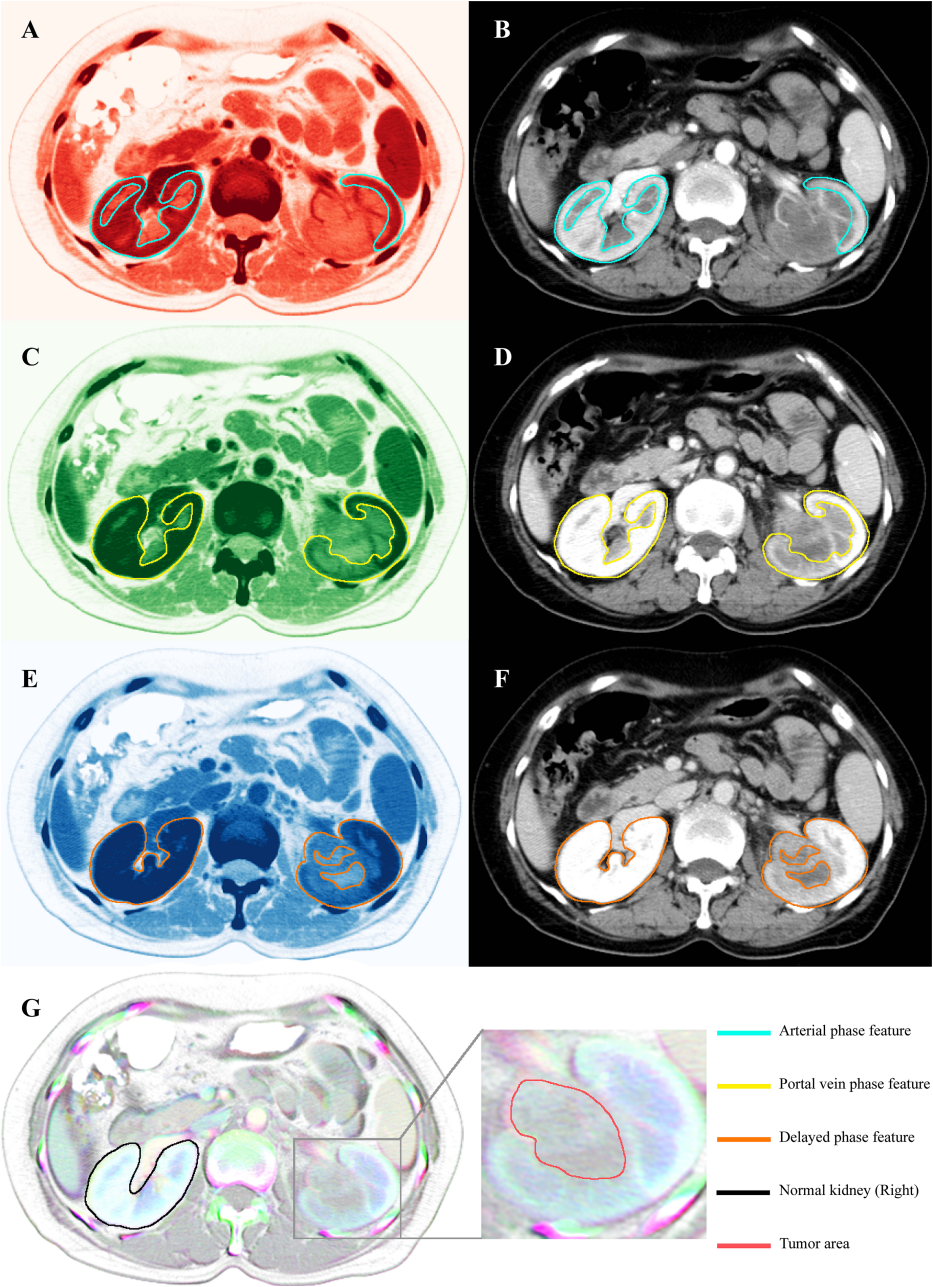
CT scan images and their corresponding features extracted for RGBA color. **(B)** Original arterial phase CT images and **(A)** arterial phase images transformed into RGBA (red) through feature extraction. The feature within the green-lined area represents the area of interest in the arterial phase. **(D)** Original portal vein phase CT images and **(C)** RGBA (green) images of the portal vein phase. The feature within the yellow-lined area represents the area of interest in the portal vein phase. **(F)** Original delayed phase CT images and **(E)** RGBA (blue) images of the delayed phase. The feature within the orange-lined area represents the area of interest in the delayed phase. **(G)** RGBA image created by the fusion of three phases of CT contrast images (red, green, and blue) and CT plain scan images (alpha). The area marked with a black line represents normal kidneys, distinguished by a white overlay of features from all channels. The enlarged section identifies the tumor region, distinguished by a gray area with central blue-green bands, which effectively captures the radiological changes across the four phases.

Each CT scan comprised four contrast-enhanced phases: plain, arterial, portal venous, and delayed. Features were initially extracted from each phase independently. Notably, during the arterial phase, enhancement of the renal cortex in the affected left kidney was markedly reduced compared to the contralateral kidney (**Figures 2A, B**). This attenuation persisted into the portal venous phase (**Figures 2C, D**). In the delayed phase, while the right kidney showed homogeneous enhancement, the medullary region of the left kidney, corresponding to the tumor site, exhibited heterogeneous enhancement (**Figures 2E, F**). These radiological features are consistent with the typical imaging patterns of UTUC, characterized by absent or reduced enhancement in early phases and partial enhancement in delayed imaging.

In the final RGBA composite image, the right kidney, consistently enhanced across arterial, portal, and delayed phases, appeared white due to full representation in the red, green, and blue channels. In contrast, the left kidney, with its heterogeneous and phase-dependent enhancement, appeared gray with central blue-green hues (**Figure 2G**). This transformation allowed for simultaneous visualization of radiologic variation across all phases, enhancing the model’s ability to capture prognostically relevant features.

These RGBA-fused images were subsequently input into the EfficientNet-B3 architecture for feature extraction and served as the imaging component of the MICC model.

### 2.5 Model architecture

We developed the MICC model by integrating multi-phase CT images with clinical information. To enable a comprehensive performance evaluation, we also constructed two single-modality models for comparison: ImageNet and ClinicalNet.

ImageNet was implemented as a binary classification model that processes RGBA-based CT images of UTUC using the EfficientNet-B3 architecture. This model directly receives multi-phase CT images as input and outputs a binary classification result, without the need for additional feature engineering. EfficientNet-B3, a state-of-the-art transfer learning network, effectively balances model depth, width, and resolution, and has demonstrated superior performance across a range of transfer learning tasks (20).

For clinical data processing, we intentionally retained all clinical features without performing explicit feature selection, based on the following considerations:

1. The sample-to-feature ratio (n/p ≈ 8.4) did not represent a typical high-dimensional, small-sample scenario;
2. The XGBoost algorithm, employed in ClinicalNet, inherently addresses feature redundancy via its tree-based structure and built-in regularization mechanisms;
3. The use of five-fold cross-validation contributed to model robustness and generalizability;
4. Preserving all clinical variables was critical for identifying potential interactions between clinical and imaging data in our multimodal fusion framework.

The ClinicalNet model was implemented using XGBoost to process the complete set of clinical features (21).

Our proposed MICC model integrates both modalities by first extracting features from CT images using EfficientNet-B3, which are then transformed into a feature vector of size 64 via a linear layer. Simultaneously, clinical features are also processed and transformed into a feature vector of size 64 using a separate linear layer. These two feature vectors are concatenated and passed through a final linear layer to generate the classification output. This fusion approach enables MICC to leverage complementary information from both imaging and clinical data.

Both the MICC and ImageNet models were trained using the cross-entropy loss function, a standard method for binary classification tasks (19, 20). For each patient, the model outputs a probability (via sigmoid activation) of being high-risk, with the loss computed as:


$$L=-\;\frac{1}{N}\sum_{\left\{i=1\right\}}^{N}\left[y_{i}\log(p_{i})+(1-y_{i})\log(1-p_{i})\right]$$

where *yi* is the true label (0 or 1), and *pi* is the predicted probability. The Adam optimizer minimizes this loss, with gradients computed based on prediction errors, ensuring efficient convergence. The ‘acc’ function calculates the model’s accuracy by determining the proportion of correctly classified samples, yielding a value between 0 and 1. To address class imbalance within the training dataset, an Imbalanced Dataset Sampler (Imbalanced Sampler) was employed. This sampler adjusts the sampling strategy to equilibrate the class distribution, preventing the model from overfitting to the majority class.

To mitigate potential data contamination and ensure an unbiased evaluation, the MICC model was trained exclusively on the Training set (n = 103). All steps involving clinical feature derivation and imaging feature extraction were performed solely on these training samples, thereby preventing any leakage of information from the Testing set. Model parameters and hyperparameter tuning were determined within the training set, and the derived features were subsequently applied to an entirely independent Testing set (n = 30) reserved for final evaluation. This strict partitioning ensures that performance metrics reflect the true generalizability of the MICC model, free from data leakage or test set contamination.

### 2.6 Model assessment

To comprehensively evaluate the diagnostic performance of each model, we considered several metrics, including accuracy, sensitivity, specificity, positive predictive value (PPV), negative predictive value (NPV), and the area under the curve (AUC). The DeLong method was used to compute the 95% confidence intervals (CIs) for the AUC. The Integrated Discrimination Improvement (IDI) was calculated using the reclassification package in R.

The Akaike Information Criterion (AIC) was determined using the formula:


AIC=2k−2ln(L)

Where *k* is the number of parameters, and *L* is the maximum likelihood estimate of the model.

The Bayesian Information Criterion (BIC) was calculated using the formula:


BIC=ln(n)k−2ln(L)

Where *n* is the sample size, *k* is the number of parameters, and *L* is the maximum likelihood estimate.

The Precision-Recall Area Under the Curve (PR AUC) was computed using the sklearn package in Python.

To explore the relevance and interactions of clinical characteristics, all patients were evaluated using the MICC model and analyzed via SHapley Additive exPlanations (SHAP) (22). SHAP, based on game theory, analyzes machine learning models by quantifying the contribution of each feature, thereby enhancing the interpretability of the model’s decision-making process.

### 2.7 Statistical analysis

All statistical calculations and graphical representations were performed using R software (version 4.1.0, https://www.r-project.org/), Python (version 3.9.7, https://www.python.org), and IBM SPSS Statistics 27 (https://www.ibm.com/spss). *P*-values were calculated using two-tailed tests, with values of *p*<0.05 considered statistically significant. The clinical characteristics of the Training and Testing groups were compared using unpaired t-tests for continuous variables and chi-squared tests for categorical variables. To assess statistical differences in accuracy, specificity, and AUC between the two models, McNemar’s Chi-squared test with continuity correction was applied. The Kaplan-Meier estimator was used to construct survival curves. Both Univariate and Multivariate Cox Regression Analyses were performed to identify independent prognostic factors.

## 3 Result

### 3.1 Patient characteristics

Details on the clinical characteristics of the patients are summarized in **Table 1**. No significant differences were observed between the Training and Testing sets (*P* > 0.05), except for tumor location and surgical method.

**Table 1:** Clinical characteristics of the UTUC patients.

Characteristic	Training cohort	Testing cohort	*p* value
	(n=103)	(n= 30)	
Sex, n(%)			0.136
Female	26 (25.24%)	12(40.00%)	
Male	77(74.76%)	18(60.00%)	
Age, n(%)			0.338
≥65 years	61(59.22%)	19(63.33%)	
<65 years	42(40.78%)	11(36.67%)	
BMI, n(%)			0.459
≥25 kg/m^2^	19(18.45%)	5(16.67%)	
<25 kg/m^2^	84(81.55%)	25(83.33%)	
ASA, grade, n(%)			0.364
≥3	28(27.18%)	5(16.67%)	
<3	75(73.82%)	25(83.33%)	
Hydronephrosis, n(%)			0.747
Yes	65(63.11%)	20(66, 67%)	
No	38(36.89%)	10(33.33%)	
HB, g/dl			0.748
Mean±SD	122.87±6.10	121.63±7.96	
Albumin, g/dl			0.215
Mean±SD	41.19±8.15	39.99±10.62	
operation method, n(%)			<0.001
Open surgery	71(68.93%)	6(20.00%)	
Laparoscopic surgery	32(31.07%)	24(80.00%)	
Tumor size, n(%)			0.959
>3 cm	37(35.92%)	15(50.00%)	
≤3 cm	66(64.03%)	15(50.00%)	
Position, n(%)			0.005
Renal pelvic carcinoma	80(77.67%)	14(46.67%)	
Ureteral carcinoma	20(19.42%)	15(50.00%)	
Both have	3(2.91%)	1(3.33%)	
Tumor number, n(%)			0.243
Multiple cancer	85(82.52%)	28(93.33%)	
Unifocal cancer	18(17.48%)	2(6.67%)	
LVI, n(%)			0.425
Yes	19(18.45%)	3(10.00%)	
No	84(81.55%)	27(90.00%)	
T stage, n(%)			0.970
pT1-pT2	61(59.22%)	18(60.00%)	
pT3-pT4	42(40.78%)	12(40.00%)	
N stage, n(%)			0.068
N0	91(88.35%)	30(100.0%)	
N1	12(11.64%)	0(0.00%)	
OS, n(%)			0.411
1 year OS rate	79.6%	96.0%	
3 years OS rate	42.7%	70.0%	
5 years OS rate	25.2%	50.0%	
10 years OS rate	1.00%	0.00%	
PFS, n(%)			0.954
1 year PFS rate	62.1%	86.7%	
3 years PFS rate	31.1%	60.0%	
5 years PFS rate	16.5%	36.7%	
CSS, n(%)			
1 year CSS rate	79.6%	96.0%	0.411
3 years CSS rate	42.7%	70.0%	
5 years CSS rate	25.2%	50.0%	
10 years CSS rate	1.00%	0.00%	

BMI, body mass index; HB, hemoglobin; LVI, Lymphovascular Invasion; OS, Overall Survival; PFS, Progression Free Survival; CSS, cancer-specific survival.

a The p-value is calculated by Chi-squared test for categorical variables. When the minimum expected count is greater than 5, use the Pearson chi-square test; when the minimum expected count is between 1 and 5, use the Yates continuity correction; when the minimum expected count is less than 1, use the Fisher's exact test. And p-value is calculated by unpaired t-tests for continuous variables.

### 3.2 Selection of the baseline network

To evaluate potential benchmark models for ImageNet, we considered EfficientNet-B3, ResNet-50, and VGG-16. These models were trained independently, and their performance was compared using both the Training and Testing sets (**Supplementary Figure 1A**). The results showed that EfficientNet-B3 outperformed the other models, achieving the highest scores for AUC (0.884), Accuracy (0.825), Sensitivity (0.667), and Specificity (0.910) on the Training set. It was followed by ResNet-50 (AUC = 0.640, Accuracy = 0.612, Sensitivity = 0.611, Specificity = 0.612), with VGG-16 exhibiting the poorest performance (AUC = 0.576, Accuracy = 0.641, Sensitivity = 0.361, Specificity = 0.791). As a result, EfficientNet-B3 was selected as the baseline network and subsequently retrained on the Training set.

### 3.3 Efficacy of the MICC model in prognostic stratification

Multimodal prediction models, which integrate multiple data sources, have been shown to offer superior predictive performance in cancer prognosis compared to models relying on a single modality (23). To test this hypothesis, we trained three distinct models using the same dataset, with varying input modalities: CT images only (ImageNet), clinical information only (ClinicalNet), and a combined approach integrating both modalities (MICC model).

**Figure 3:**
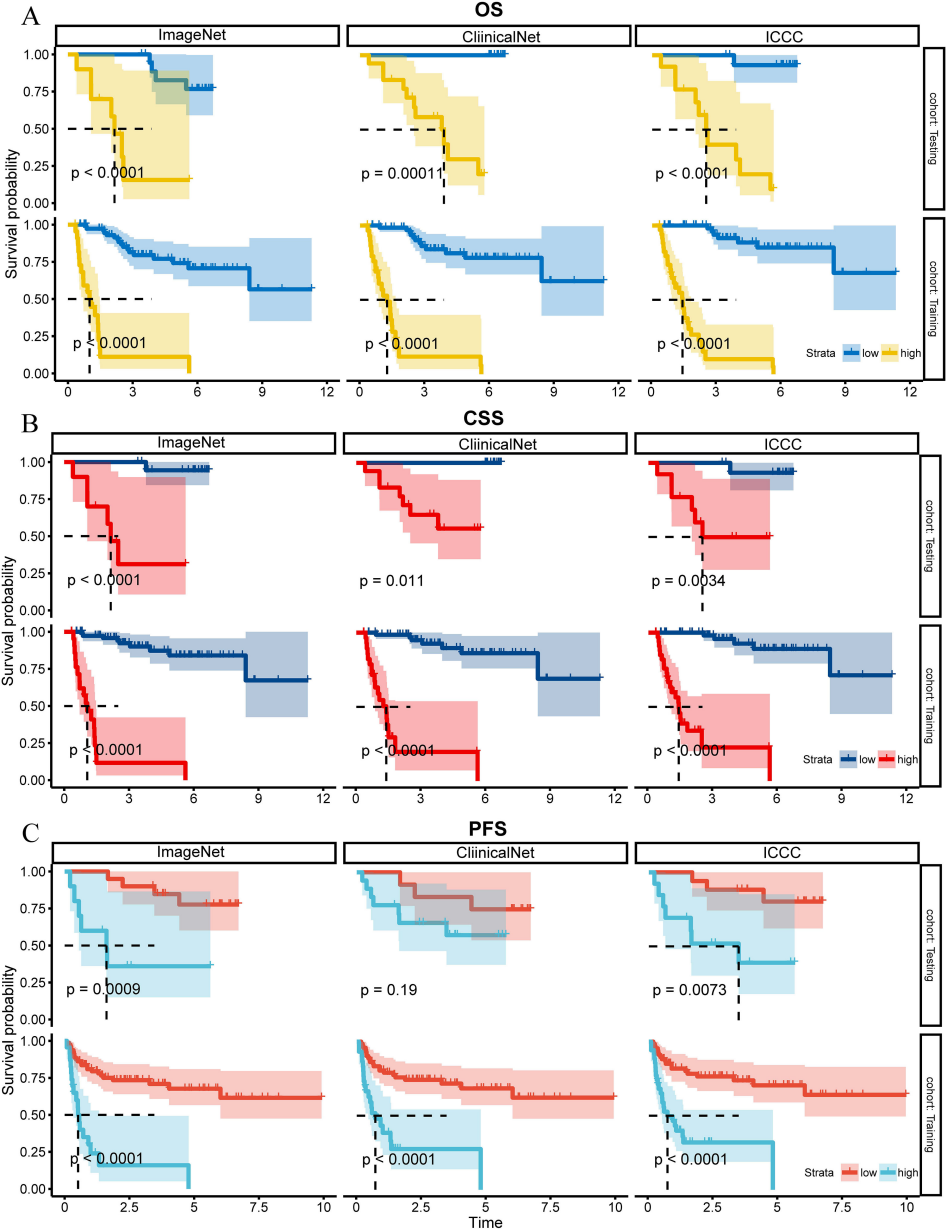
K-M curves for OS, CSS, and PFS for ClinicalNet, ImageNet, and MICC model in Training and Testing sets. **(A)** K-M curves for OS, **(B)** K-M curves for CSS, and **(C)** K-M curves for PFS, comparing ClinicalNet, ImageNet, and MICC model across both settings.

Kaplan-Meier survival curves confirmed that all three models were effective in differentiating between high-risk and low-risk patients in both the Training and Testing sets with regard to OS, CSS, and PFS (**Figure 3**, *P*< 0.05), although the ClinicalNet model did not demonstrate significant predictive ability for PFS in the Testing set (*P* = 0.19).

### 3.4 Discriminative and calibration performance of the MICC model

**Figure 4:**
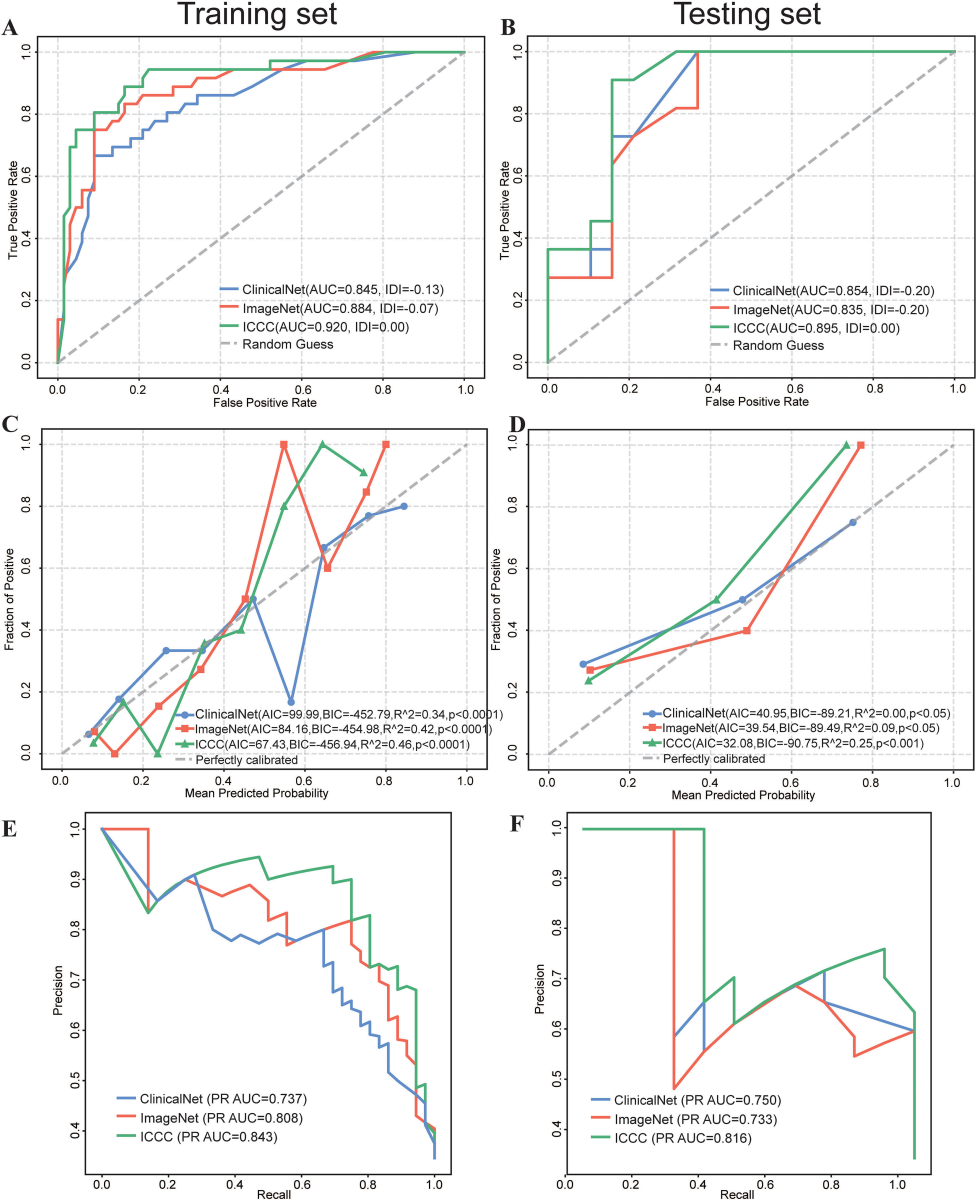
Comparative Performance of ClinicalNet, ImageNet, and MICC Model. **(A)** ROC curves and IDI scores for the three models in the Training set. **(B)** ROC curves and IDI scores in the Testing set. **(C)** Calibration curves, along with AIC, BIC, and R^^^2 values, assess the predictive accuracy of the three models in the Training set, grouping every 10 patients. **(C)** Calibration curves, along with AIC, BIC, and R^^^2 values, assess the predictive accuracy of the three models in the Testing set **(E)** PR Curve assessing each model’s ability to identify patients at risk of death in the Training set. **(F)** PR Curve assessing each model’s ability to identify patients at risk of death in the Testing set.

Receiver Operating Characteristic (ROC) analysis revealed that the MICC model consistently outperformed ImageNet and ClinicalNet in both the Training and Testing sets. In the Training set, MICC achieved an AUC of 0.918, surpassing ImageNet (AUC = 0.884) and ClinicalNet (AUC = 0.845). Similarly, in the Testing set, MICC maintained the highest performance with an AUC of 0.895, followed by ImageNet (AUC = 0.835) and ClinicalNet (AUC = 0.854). Furthermore, when MICC was used as the benchmark, the Integrated Discrimination Improvement (IDI) values for both ImageNet and ClinicalNet were negative, indicating a decline in their predictive performance relative to MICC (**Figures 4A, B**).

Calibration curves were employed to assess the predictive accuracy of the three models, and results indicated that all models performed robustly. In the Testing set, MICC demonstrated the best calibration performance, with the lowest AIC (67.43) and BIC (–456.94), as well as the highest R² value of 0.46 (p< 0.0001), indicating an optimal balance between model fit and complexity (**Figure 4C**). In the Training set, MICC predictions were closest to the ideal calibration line across all probability bins, further underscoring its superior ability to generate reliable and well-calibrated risk estimates (**Figure 4D**). These results highlight the advantage of integrating multimodal data to improve prognostic accuracy in clinical settings.

### 3.5 Precision–recall analysis and predictive score distribution

Given the small proportion of actual deaths in our dataset, early identification of high-risk patients is crucial. To address this, we utilized the precision-recall (PR) curve to evaluate the performance of the three models. The PR curve is particularly valuable for assessing the ability to recognize positive class samples (deaths) within an imbalanced dataset.

In the Training set, the results demonstrated that the MICC model excelled in predicting deaths, achieving the highest PR AUC of 0.843 (**Figure 4E**), followed by ImageNet (PR AUC = 0.808) and ClinicalNet (PR AUC = 0.737). Similar results were observed in the Testing set, where MICC also achieved the highest PR AUC of 0.816 (**Figure 4F**).

The distribution of prediction scores for the three models in the Training set, shown in raincloud plots, reveals that MICC’s predictions are the most narrowly distributed and closely align with a normal distribution (**Supplementary Figure 1B**).

### 3.6 Model evaluation of classification performance metrics

A comprehensive evaluation of the three models demonstrated significant improvements with the MICC model in the Training set, including accuracy (0.854, 95% CI = 0.852–0.857), sensitivity (0.889, 95% CI = 0.786–0.992), NPV (0.933, 95% CI = 0.870–0.996), and AUC (0.920, 95% CI = 0.860–0.979) (**Table 2**, **Supplementary Figure 1C**). In the Testing set, the MICC model maintained notably stable performance, with all indicators closely aligning with those of the Training set: accuracy (0.867, 95% CI = 0.859–0.874), sensitivity (0.909, 95% CI = 0.739–1.000), specificity (0.842, 95% CI = 0.678–1.000), PPV (0.769, 95% CI = 0.540–0.998), NPV (0.941, 95% CI = 0.829–1.000), and AUC (0.895, 95% CI = 0.780–1.000).

**Table 2:** Performance of the TestNet, ImageNet and ITCC model on the Training and Testing cohort.

Training cohort Metric	Model		
	ClinicalNet Model	ImageNet Model	ICCC Model
Accuracy	0.825(0.822-0.828)	0.835(0.832-0.838)	0.854(0.852-0.857)
Sensitivity	0.667(0.513-0.821)	0.833(0.712-0.955)	0.889(0.786-0.992)
Specificity	0.910(0.842-0.979)	0.836(0.747-0.925)	0.836(0.747-0.925)
PPV	0.800(0.657-0.943)	0.732(0.596-0.867)	0.744(0.614-0.875)
NPV	0.836(0.751-0.921)	0.903(0.830-0.977)	0.933(0.870-0.996)
AUC	0.845(0.765-0.924)	0.884(0.814-0.954)	0.920(0.860-0.979)
Training cohort Metric	Model		
	ClinicalNet Model	ImageNet Model	ICCC Model
Accuracy	0.767(0.755-0.778)	0.767(0.755-0.778)	0.867(0.859-0.874)
Sensitivity	1.000(1.000-1.000)	0.636(0.316-0.876)	0.909(0.739-1.000)
Specificity	0.632(0.415-0.848)	0.842(0.595-0.958)	0.842(0.678-1.000)
PPV	0.611(0.386-0.836)	0.700(0.354-0.919)	0.769(0.540-0.998)
NPV	1.000(1.000-1.000)	0.800(0.557-0.934)	0.941(0.829-1.000)
AUC	0.854(0.721-0.988)	0.835(0.692-0.978)	0.895(0.780-1.000)

PPV, positive predictive value; NPV, negative predictive value; AUC, area under the Receiver Operating Characteristic curve.

data in parentheses are 95% confidence intervals (95%CI), calculated by Wald Z Method with Continuity Correction for accuracy, sensitivity specificity, PPV and NPV, and by DeLong method for AUC.

In contrast, the performance of ClinicalNet varied substantially between the Training and Testing sets, indicating limited generalizability and robustness. Furthermore, its relatively low sensitivity in the Training set (0.667, 95% CI = 0.513–0.821) suggests potential difficulties in timely identifying high-risk patients, which could hinder early clinical intervention.

### 3.7 Analysis of feature importance in the MICC model

**Figure 5:**
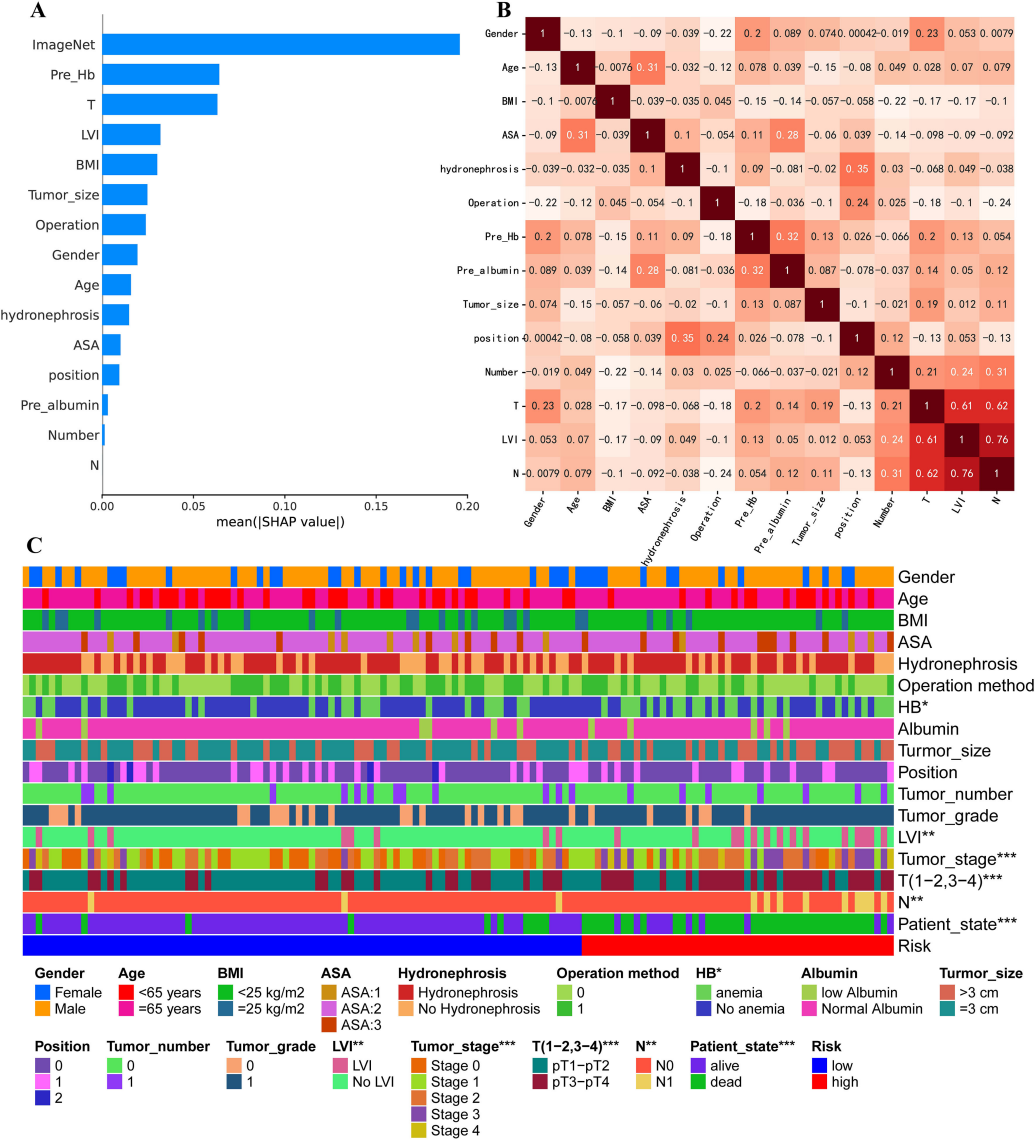
Comparison of clinical feature importance in Training set. **(A)** Ranking of contributing factors to the MICC model in Training set, obtained using the SHAP method. **(B)** Pearson correlation coefficient analysis for all clinical factors. **(C)** Heat map comparing all clinical characteristics between high-risk and low-risk groups.

To identify the key contributors to the predictive performance of the MICC model, we conducted SHAP analysis on the Training set. Among all features, ImageNet emerged as the most influential variable, followed by preoperative hemoglobin (HB), T stage, and Lymphovascular invasion (LVI), highlighting the dominant role of imaging-derived information in the multimodal framework (**Figure 5A**). Similarly, in Testing set, ImageNet remained the most influential variable (**Supplementary Figure 1D**).

To assess potential feature redundancy, we performed Pearson correlation analysis among the clinical variables included in the MICC model. All features were largely independent (correlation< 0.5), except for a moderate correlation observed between T stage, LVI, and N stage (**Figure 5B**). Furthermore, we observed significant differences in key clinical features—including preoperative HB, LVI, T stage, and N stage—between high-risk and low-risk groups, further supporting their prognostic relevance within the model (**Figure 5C**).

## 4 Discussion

In this study, we developed a multimodal DL model, named the MICC model, to predict postoperative survival for patients with UTUC using multi-phase CT images and clinical data. Our findings demonstrate that, the MICC model surpassed both ImageNet and ClinicalNet, yielding AUCs of 0.918 in training and 0.895 in testing. It retained strong accuracy (0.854), sensitivity (0.889), specificity (0.836), NPV (0.933), and PPV (0.744), while precision-recall analysis confirmed its superior ability to detect high-risk patients in an imbalanced cohort. These gains arise from fusing four-phase CT scans into RGBA composites, which captured subtle enhancement dynamics overlooked by conventional approaches; SHAP analysis further showed that these CT-derived signals dominate the model’s prognostic predictions.

Recent advancements in DL have significantly influenced oncological research. For instance, Y. Wang et al. utilized DL to enhance histological grading of breast cancer (24), and Y. Jiang et al. applied DL to assess the tumor microenvironment of gastric cancer and predict treatment responses (25). These studies often involved training models from scratch using frameworks such as convolutional neural networks (CNN), which present several limitations, including the potential waste of a large number of samples and restricted model generalization, particularly when relying on a single training dataset (26). In contrast, our study employed transfer learning, which enabled superior performance with fewer samples. Transfer learning enhanced the robustness of the MICC model, making it particularly effective when applied to new datasets with varying distributions (Training set AUC = 0.918; Testing set AUC = 0.895).

Our MICC model demonstrates several distinct advantages over existing UTUC prognostic approaches. Compared to traditional radiomics methods that require manual ROI delineation and extract thousands of hand-crafted features, our RGBA fusion approach enables automated end-to-end feature learning while preserving complete multi-phase temporal information. Unlike single-phase deep learning approaches that lose critical contrast dynamics, our method captures the full spectrum of enhancement patterns across all four phases. Furthermore, our multimodal fusion strategy surpasses conventional statistical methods by automatically learning complex non-linear interactions between imaging and clinical features, eliminating the need for explicit feature selection and dimensionality reduction steps that may introduce bias or information loss.

Our MICC model achieved exceptional prognostic performance with AUC values of 0.918 and 0.895 in training and testing sets, respectively, representing a significant advancement over traditional UTUC prognostic approaches. Compared to conventional statistical methods represented by our ClinicalNet model (AUC = 0.854), our deep learning approach demonstrates superior predictive capability. The model’s high sensitivity (0.889) and negative predictive value (0.933) are particularly valuable for early identification of high-risk patients, enabling timely intervention and personalized treatment planning. Our study reveals several important prognostic insights that distinguish it from existing research. First, SHAP analysis demonstrated that imaging features contribute most significantly to prognostic predictions, surpassing traditional clinical pathological variables in importance. This finding provides a new perspective for UTUC prognostic assessment, suggesting that multi-phase CT imaging contains prognostic information beyond conventional clinical evaluation. The superiority of our multi-modal MICC model (AUC = 0.895) over the purely clinical ClinicalNet model (AUC = 0.854) provides direct evidence for the independent prognostic value of preoperative imaging features. Second, our identification of preoperative hemoglobin levels as an independent prognostic factor complements existing literature while providing clinicians with an additional, easily accessible prognostic tool. Third, the successful integration of temporal contrast dynamics through RGBA fusion enables identification of tumor vascularization patterns and enhancement heterogeneity, which are critical prognostic indicators not captured by traditional single-phase analyses. These dynamic enhancement patterns reflect underlying tumor biology including angiogenesis, cellular density, and stromal composition, which are fundamental determinants of tumor aggressiveness and prognosis. The consistent ranking of imaging features as the most important prognostic factor across both training and testing sets validates the robustness of this finding and supports the clinical utility of preoperative imaging in UTUC prognostic assessment. These findings collectively advance our understanding of UTUC prognosis and provide a robust foundation for clinical decision making.

Moreover, the MICC model leveraged a multimodal approach that integrated multi-phase CT images and clinical data, improving diagnostic precision. This integration resulted in better AIC and BIC scores compared to the ClinicalNet and ImageNet models. These results underscore the advantages of DL in maximizing data utilization. Our findings suggest that combining multi-phase CT imagery with clinical information through DL offers a promising pathway for enhancing prognostic assessments in cancer studies.

The ClinicalNet, which was constructed using only clinical information, demonstrated limited performance in differentiating patients’ PFS, highlighting the limitations of relying solely on clinical data for practical clinical applications. This underscores the necessity of integrating a broader spectrum of patient data to improve prognostic accuracy. CT imaging features play a crucial role in diagnosing UTUC (27). In our study, ImageNet emerged as a significant contributor within the MICC model, emphasizing the critical role of CT imaging in the prognosis of UTUC patients. However, research on the comprehensive use of CT imaging in this context remains relatively scarce.

In our approach, we employed RGBA images through a multi-sequence fusion method to capture dynamic changes across four-phase CT scans, which resulted in excellent performance. This method highlights that subtle variations in CT image characteristics, especially those evident between different phases of CT imaging, are integral to the prognostic assessment of UTUC patients. Future research should focus more on the importance of these subtle CT image details to further enhance the accuracy of prognostic evaluations for UTUC patients.

Among clinical factors, preoperative HB, LVI, and T stage are recognized as significant prognostic indicators. T stage and LVI have been widely acknowledged as potent prognostic factors in numerous studies and are frequently incorporated into various UTUC prognostic models (28, 29). Notably, the association between preoperative HB and UTUC prognosis has not been extensively explored. Anemia often manifests in advanced cancer cases, and many UTUC patients suffer from this condition (30). Teruo Inamoto et al. proposed that preoperative HB may reflect biological age, influencing CSS in UTUC patients (31). Similarly, Dong Fang et al. identified preoperative anemia as a crucial prognostic factor for predicting postoperative metastasis and CSS in UTUC cases (32). Research suggests that UTUC may induce anemia by impairing the body’s ability to utilize iron (33, 34), disrupting normal kidney function, and reducing erythropoietin production in the kidneys (35). Consequently, anemia not only serves as a hematological marker for diagnosing UTUC but also indicates poor prognosis, with anemic patients at increased risk of recurrence and metastasis. These findings underscore the importance of preoperative HB and advocate for its incorporation into treatment planning for UTUC patients.

Urothelial carcinoma, as a continuous disease spectrum, encompasses upper tract urothelial carcinoma (UTUC) and lower tract urothelial carcinoma. Despite their histological homology, they exhibit significant differences in epidemiology, biological behavior, and diagnostic and therapeutic strategies. Among them, bladder cancer, as the most common type of lower tract urothelial carcinoma, often leads advancements in the entire field of urothelial carcinoma. In recent years, breakthroughs have been made in the neoadjuvant therapy of bladder cancer, particularly in immunotherapy and targeted therapy. For example, the multicenter real-world study by Hu, Jiao et al. demonstrated the outstanding efficacy of neoadjuvant disitamab vedotin (RC48-ADC) combined with immunotherapy in muscle-invasive bladder cancer (MIBC) (36). Through multi-omics analysis, the study revealed the value of HER2 expression and specific immune microenvironment markers as predictors of therapeutic response, advancing precision medicine. A prior retrospective study also systematically confirmed the advantages of neoadjuvant immunotherapy, chemotherapy, and combination therapy in MIBC (37). However, the diagnosis and treatment of UTUC face unique challenges, such as reliance on imaging for preoperative diagnosis and the urgent need for kidney preservation, making its therapeutic strategies distinct from those of bladder cancer. Drawing on the successful application of multi-omics technologies (genomics, transcriptomics, etc.) in bladder cancer, this strategy also holds promise for optimizing precision therapy in UTUC.

This study focuses on UTUC, where the developed RGBA fusion technology and multimodal deep learning methods not only serve prognostic prediction and individualized treatment decision-making for UTUC but also possess a universal framework with the potential to extend to bladder cancer imaging analysis. Based on the successful experience in bladder cancer treatment, future research should prioritize: leveraging bladder cancer insights to develop neoadjuvant regimens for UTUC, constructing UTUC-specific multi-omics databases to identify biomarkers, and developing AI-powered diagnostic and therapeutic platforms that integrate multimodal information.

Our study is distinguished by several key advantages. First, we introduced a novel RGBA-based method to consolidate the four distinct phases of CT images into a single input for the DL model. This approach effectively captures the temporal dynamics of contrast-enhanced CT images, while avoiding the information loss typically associated with conventional image fusion or selection techniques (38). Second, we employed EfficientNet-B3 as the baseline network for extracting features from CT images. EfficientNet is a state-of-the-art DL architecture known for its ability to optimally balance depth, width, and resolution, resulting in superior performance across various transfer learning tasks. Third, our department, the largest urologic center in southern Zhejiang Province, boasts the largest sample size of UTUC patients in the region. This extensive sample size allows our findings to more accurately reflect real-world scenarios, enhancing the validity and applicability of our model while minimizing risks associated with overfitting or selection bias.

While our study presents significant advancements in UTUC prognosis prediction, several limitations must be acknowledged. First, the retrospective design introduces potential selection bias and limits causal inference, with retrospective data collection potentially resulting in incomplete information compared to prospective studies. Second, our single center design may limit generalizability to populations with different demographic characteristics, imaging protocols, or clinical practices. Third, although our cohort represents the largest UTUC dataset in our region (n=133), the sample size remains relatively small for deep learning applications, particularly for the testing set (n=30), potentially limiting statistical power and model stability. Fourth, the 12-year temporal span (2005-2017) may introduce bias due to evolving surgical techniques, imaging protocols, and clinical management practices. Fifth, our model utilized only single axial slices rather than full 3D volumetric analysis, potentially missing complete spatial tumor heterogeneity. Sixth, the lack of external validation limits assessment of true generalizability and clinical applicability. Seventh, our innovative RGBA fusion approach requires further validation against established radiomics methods. Finally, some potentially important prognostic factors (molecular markers, genetic profiles, detailed histological subtypes) were not included due to data availability constraints. Future studies should address these limitations through prospective multicenter validation, larger diverse patient populations, 3D volumetric analysis, molecular biomarker integration, and extended follow-up periods.

## 5 Conclusion

We have developed a DL model that integrates multi-phase CT images and clinical data for the prognostic assessment of UTUC patients. This model shows great potential in assisting physicians with personalized treatment strategies, ultimately enhancing the overall prognosis for UTUC patients.

## Data Availability

The raw data supporting the conclusions of this article will be made available by the authors, without undue reservation.

## Supplementary material

Supplementary Figure 1 EfficientNet-B3 was chosen as the baseline network. **(A)** The performance of Efficientnet-B3, ResNet-50, and VGG-16 in the Training and Testing sets is presented. Evaluation metrics include AUC, Accuracy, Sensitivity, and Specificity. **(B)** Raincloud plot illustrating the distribution of predicted values for ClinicalNet, ImageNet, and MICC model. **(C)** Performance comparison of ClinicalNet, ImageNet, and MICC model based on accuracy, specificity, and AUC in Training set. The p-value is calculated using McNemar’s Chi-squared test with continuity correction. Error bars indicate 95% CIs, calculated using the Wald Z Method with Continuity Correction for accuracy and specificity, and the DeLong method for AUC. **(D)** Ranking of contributing factors to the MICC model in Testing set, obtained using the SHAP method.

## References

[1] Siegel RL Miller KD Jemal A. Cancer statistics, 2020. CA Cancer J Clin. (2020) 70:7–30. doi: 10.3322/caac.21590, PMID: 31912902

[2] Soria F Shariat SF Lerner SP Fritsche HM Rink M Kassouf W. Epidemiology, diagnosis, preoperative evaluation and prognostic assessment of upper-tract urothelial carcinoma (UTUC). World J Urol. (2017) 35:379–87. doi: 10.1007/s00345-016-1928-x, PMID: 27604375

[3] Yakoubi R Colin P Seisen T Léon P Nison L Bozzini G. Radical nephroureterectomy versus endoscopic procedures for the treatment of localised upper tract urothelial carcinoma: a meta-analysis and a systematic review of current evidence from comparative studies. Eur J Surg Oncol. (2014) 40:1629–34. doi: 10.1016/j.ejso.2014.06.007, PMID: 25108813

[4] Bao Z Zhan Y He S Li Y Guan B He Q. Increased expression of SOX2 predicts A poor prognosis and promotes Malignant phenotypes in upper tract urothelial carcinoma. Cancer Manag Res. (2019) 11:9095–106. doi: 10.2147/CMAR.S219568, PMID: 31695499 PMC6817346

[5] Kramer A Pippias M Noordzij M Stel VS Afentakis N Ambühl PM. The European renal association - European dialysis and transplant association (ERA-EDTA) registry annual report 2015: a summary. Clin Kidney J. (2018) 11:108–22. doi: 10.1093/ckj/sfx149, PMID: 29423210 PMC5798130

[6] Froemming A Potretzke T Takahashi N Kim B. Upper tract urothelial cancer. Eur J Radiol. (2018) 98:50–60. doi: 10.1016/j.ejrad.2017.10.021, PMID: 29279170

[7] Millán-Rodríguez F Palou J de la Torre-Holguera P Vayreda-Martija JM Villavicencio-Mavrich H Vicente-Rodríguez J. Conventional CT signs in staging transitional cell tumors of the upper urinary tract. Eur Urol. (1999) 35:318–22. doi: 10.1159/000019869, PMID: 10087395

[8] Wang R Dai W Gong J Huang M Hu T Li H. Development of a novel combined nomogram model integrating deep learning-pathomics, radiomics and immunoscore to predict postoperative outcome of colorectal cancer lung metastasis patients. J Hematol Oncol. (2022) 15:11. doi: 10.1186/s13045-022-01225-3, PMID: 35073937 PMC8785554

[9] Foersch S Glasner C Woerl AC Eckstein M Wagner DC Schulz S. Multistain deep learning for prediction of prognosis and therapy response in colorectal cancer. Nat Med. (2023) 29:430–9. doi: 10.1038/s41591-022-02134-1, PMID: 36624314

[10] She Y Jin Z Wu J Deng J Zhang L Su H. Development and validation of a deep learning model for non-small cell lung cancer survival. JAMA Netw Open. (2020) 3:e205842. doi: 10.1001/jamanetworkopen.2020.5842, PMID: 32492161 PMC7272121

[11] Chaudhary K Poirion OB Lu L Garmire LX. Deep learning-based multi-omics integration robustly predicts survival in liver cancer. Clin Cancer Res. (2018) 24:1248–59. doi: 10.1158/1078-0432.CCR-17-0853, PMID: 28982688 PMC6050171

[12] Pallauf M König F D'Andrea D Laukhtina E Mostafaei H Motlagh RS. A systematic review and meta-analysis of prognostic nomograms after UTUC surgery. Front Oncol. (2022) 12:907975. doi: 10.3389/fonc.2022.907975, PMID: 35847838 PMC9283688

[13] Gayap HT Akhloufi MA. Deep machine learning for medical diagnosis, application to lung cancer detection: A review. BioMedInformatics. (2024) 4:236–84. doi: 10.3390/biomedinformatics4010015

[14] Zhao X Liang YJ Zhang X Wen DX Fan W Tang LQ. Deep learning signatures reveal multiscale intratumor heterogeneity associated with biological functions and survival in recurrent nasopharyngeal carcinoma. Eur J Nucl Med Mol Imaging. (2022) 49:2972–82. doi: 10.1007/s00259-022-05793-x, PMID: 35471254

[15] Alqahtani A Bhattacharjee S Almopti A Li C Nabi G. Radiomics-based machine learning approach for the prediction of grade and stage in upper urinary tract urothelial carcinoma: a step towards virtual biopsy. Int J Surg. (2024) 110:3258–68. doi: 10.1097/JS9.0000000000001483, PMID: 38704622 PMC11175789

[16] Xiao B Lv Y Peng C Wei Z Xv Q Lv F. Deep learning feature-based model for predicting lymphovascular invasion in urothelial carcinoma of bladder using CT images. Insights into Imaging. (2025) 16:108. doi: 10.1186/s13244-025-01988-6, PMID: 40382748 PMC12086130

[17] Zappia J Yong C Slaven J Wu Z Wang L Djaladat H. Survival outcomes by race following surgical treatment for upper tract urothelial carcinoma. Clin Genitourinary Cancer. (2024) 22:102220. doi: 10.1016/j.clgc.2024.102220, PMID: 39332082

[18] Zheng Y Shi H Fu S Wang H Wang J Li X. A computed tomography urography-based machine learning model for predicting preoperative pathological grade of upper urinary tract urothelial carcinoma. Cancer Med. (2024) 13:e6901. doi: 10.1002/cam4.6901, PMID: 38174830 PMC10807597

[19] Gao R Zhao S Aishanjiang K Cai H Wei T Zhang Y. Deep learning for differential diagnosis of Malignant hepatic tumors based on multi-phase contrast-enhanced CT and clinical data. J Hematol Oncol. (2021) 14:154. doi: 10.1186/s13045-021-01167-2, PMID: 34565412 PMC8474892

[20] Tan M Le Q. Efficientnet: Rethinking model scaling for convolutional neural networks. Int Conf Mach learning. (2019) 6105–14. doi: 10.48550/arXiv.1905.11946

[21] Chen T Guestrin C. XGBoost: A scalable tree boosting system, in: Proceedings of the 22nd ACM SIGKDD International Conference on Knowledge Discovery and Data Mining, (San Francisco, California, USA: KDD ’16). (2016) 785–94.

[22] Lundberg SM Erion G Chen H DeGrave A Prutkin JM Nair B. From local explanations to global understanding with explainable AI for trees. Nat Mach Intell. (2020) 2:56–67. doi: 10.1038/s42256-019-0138-9, PMID: 32607472 PMC7326367

[23] Steyaert S Qiu YL Zheng Y Mukherjee P Vogel H Gevaert O. Multimodal deep learning to predict prognosis in adult and pediatric brain tumors. Commun Med (Lond). (2023) 3:44. doi: 10.1038/s43856-023-00276-y, PMID: 36991216 PMC10060397

[24] Wang Y Acs B Robertson S Liu B Solorzano L Wählby C. Improved breast cancer histological grading using deep learning. Ann Oncol. (2022) 33:89–98. doi: 10.1016/j.annonc.2021.09.007, PMID: 34756513

[25] Jiang Y Zhou K Sun Z Wang H Xie J Zhang T. Non-invasive tumor microenvironment evaluation and treatment response prediction in gastric cancer using deep learning radiomics. Cell Rep Med. (2023) 4:101146. doi: 10.1016/j.xcrm.2023.101146, PMID: 37557177 PMC10439253

[26] Morid MA Borjali A Del Fiol G. A scoping review of transfer learning research on medical image analysis using ImageNet. Comput Biol Med. (2021) 128:104115. doi: 10.1016/j.compbiomed.2020.104115, PMID: 33227578

[27] Baard J de Bruin DM Zondervan PJ Kamphuis G de la Rosette J Laguna MP. Diagnostic dilemmas in patients with upper tract urothelial carcinoma. Nat Rev Urol. (2017) 14:181–91. doi: 10.1038/nrurol.2016.252, PMID: 27958391

[28] Favaretto RL Shariat SF Savage C Godoy G Chade DC Kaag M. Combining imaging and ureteroscopy variables in a preoperative multivariable model for prediction of muscle-invasive and non-organ confined disease in patients with upper tract urothelial carcinoma. BJU Int. (2012) 109:77–82. doi: 10.1111/j.1464-410X.2011.10288.x, PMID: 21631698 PMC4319659

[29] Zhang GL Zhou W. A model for the prediction of survival in patients with upper tract urothelial carcinoma after surgery. Dose Response. (2019) 17:1559325819882872. doi: 10.1177/1559325819882872, PMID: 31662711 PMC6794662

[30] Steinberg D. Anemia and cancer. CA Cancer J Clin. (1989) 39:296–304. doi: 10.3322/canjclin.39.5.296, PMID: 2513101

[31] Inamoto T Matsuyama H Ibuki N Komura K Fujimoto K Shiina H. Risk stratification by means of biological age-related factors better predicts cancer-specific survival than chronological age in patients with upper tract urothelial carcinoma: a multi-institutional database study. Ther Adv Urol. (2018) 10:403–10. doi: 10.1177/1756287218811050, PMID: 30574200 PMC6295779

[32] Fang D Singla N Bao Z Jafri SM Su X Cao Z. The significance of preoperative serum sodium and hemoglobin in outcomes of upper tract urothelial carcinoma: multi-center analysis between China and the United States. Cancer Manag Res. (2020) 12:9825–36. doi: 10.2147/CMAR.S267969, PMID: 33116841 PMC7549885

[33] Gilreath JA Stenehjem DD Rodgers GM. Diagnosis and treatment of cancer-related anemia. Am J Hematol. (2014) 89:203–12. doi: 10.1002/ajh.23628, PMID: 24532336

[34] Yeh HC Chien TM Wu WJ Li CC Li WM Ke HL. Is preoperative anemia a risk factor for upper tract urothelial carcinoma following radical nephroureterectomy? Urol Oncol. (2016) 34:337 e1–9. doi: 10.1016/j.urolonc.2016.03.018, PMID: 27133951

[35] Gaspar BL Sharma P Das R. Anemia in Malignancies: pathogenetic and diagnostic considerations. Hematology. (2015) 20:18–25. doi: 10.1179/1607845414Y.0000000161, PMID: 24666207

[36] Hu J Yan L Liu J Chen M Liu P Deng D. Efficacy and biomarker analysis of neoadjuvant disitamab vedotin (RC48-ADC) combined immunotherapy in patients with muscle-invasive bladder cancer: A multi-center real-world study. iMeta. (2025) 4(3):e70033. doi: 10.1002/imt2.70033, PMID: 40469503 PMC12130573

[37] Hu J Chen J Ou Z Chen H Liu Z Chen M. Neoadjuvant immunotherapy, chemotherapy, and combination therapy in muscle invasive bladder cancer: A multi-center real-world retrospective study. Cell Rep Med. (2022) 3:100785. doi: 10.1016/j.xcrm.2022.100785, PMID: 36265483 PMC9729796

[38] Liu Z Miao J Huang P Wang W Wang X Zhai Y. A deep learning method for producing ventilation images from 4DCT: First comparison with technegas SPECT ventilation. Med Phys. (2020) 47:1249–57. doi: 10.1002/mp.14004, PMID: 31883382

