# Supplementary figures and images for: A multi-data fusion deep learning model for prognostic prediction in upper tract urothelial carcinoma
Source: Front Oncol. 2025 Aug 6;15:1644250. doi: 10.3389/fonc.2025.1644250 (PMC12364636; doi:10.3389/fonc.2025.1644250)

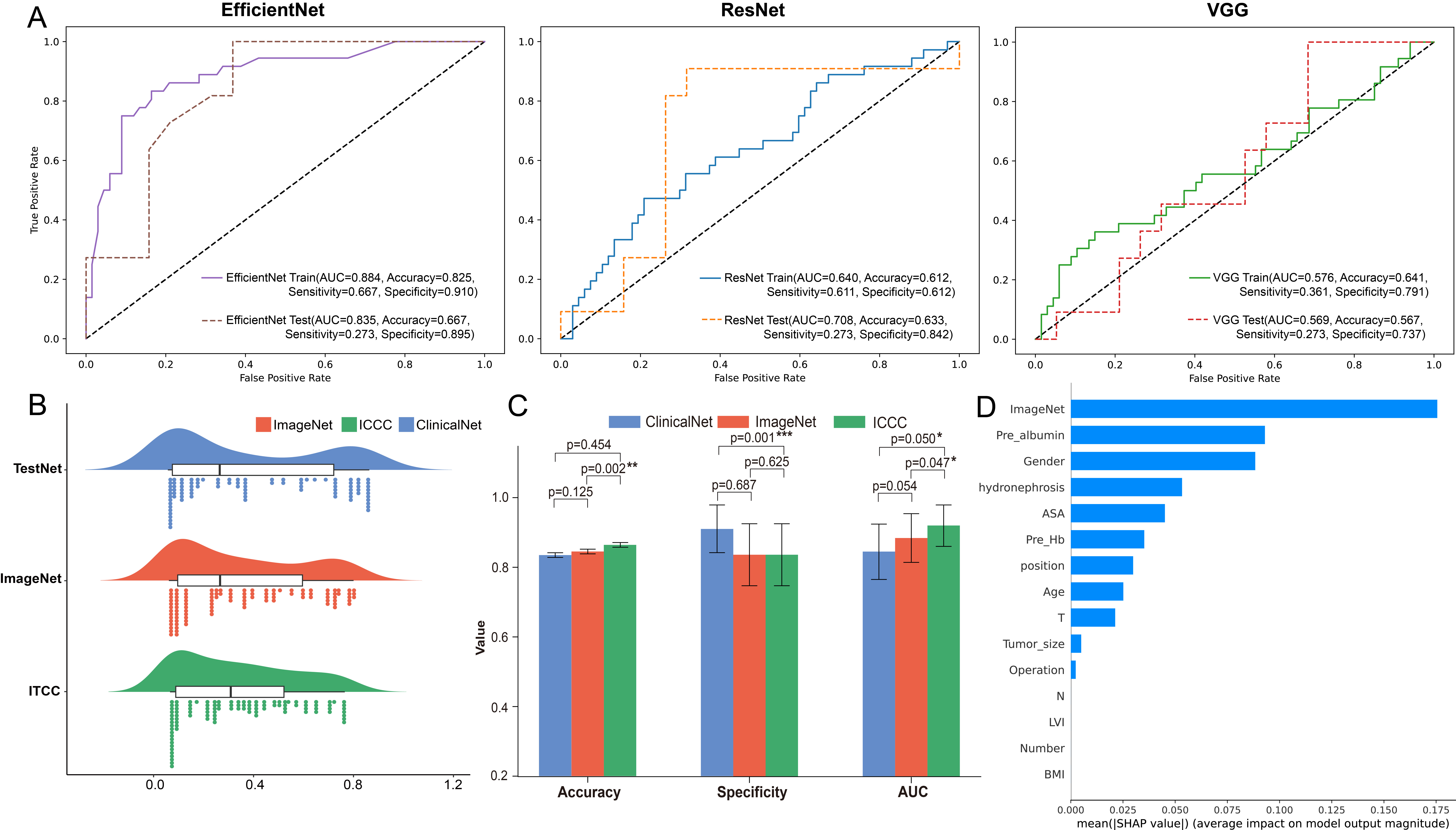

Supplement: Supplementary Figure 1 — EfficientNet-B3 was chosen as the baseline network. (A) The performance of Efficientnet-B3, ResNet-50, and VGG-16 in the Training and Testing sets is presented. Evaluation metrics include AUC, Accuracy, Sensitivity, and Specificity. (B) Raincloud plot illustrating the distribution of predicted values for ClinicalNet, ImageNet, and MICC model. (C) Performance comparison of ClinicalNet, ImageNet, and MICC model based on accuracy, specificity, and AUC in Training set. The p-value is calculated using McNemar’s Chi-squared test with continuity correction. Error bars indicate 95% CIs, calculated using the Wald Z Method with Continuity Correction for accuracy and specificity, and the DeLong method for AUC. (D) Ranking of contributing factors to the MICC model in Testing set, obtained using the SHAP method. [file Image1.jpeg]
